# Supplementary figures and images for: Brachyury cooperates with Wnt/β-catenin signalling to elicit primitive-streak-like behaviour in differentiating mouse embryonic stem cells
Source: BMC Biol. 2014 Aug 13;12:63. doi: 10.1186/s12915-014-0063-7 (PMC4171571; doi:10.1186/s12915-014-0063-7)

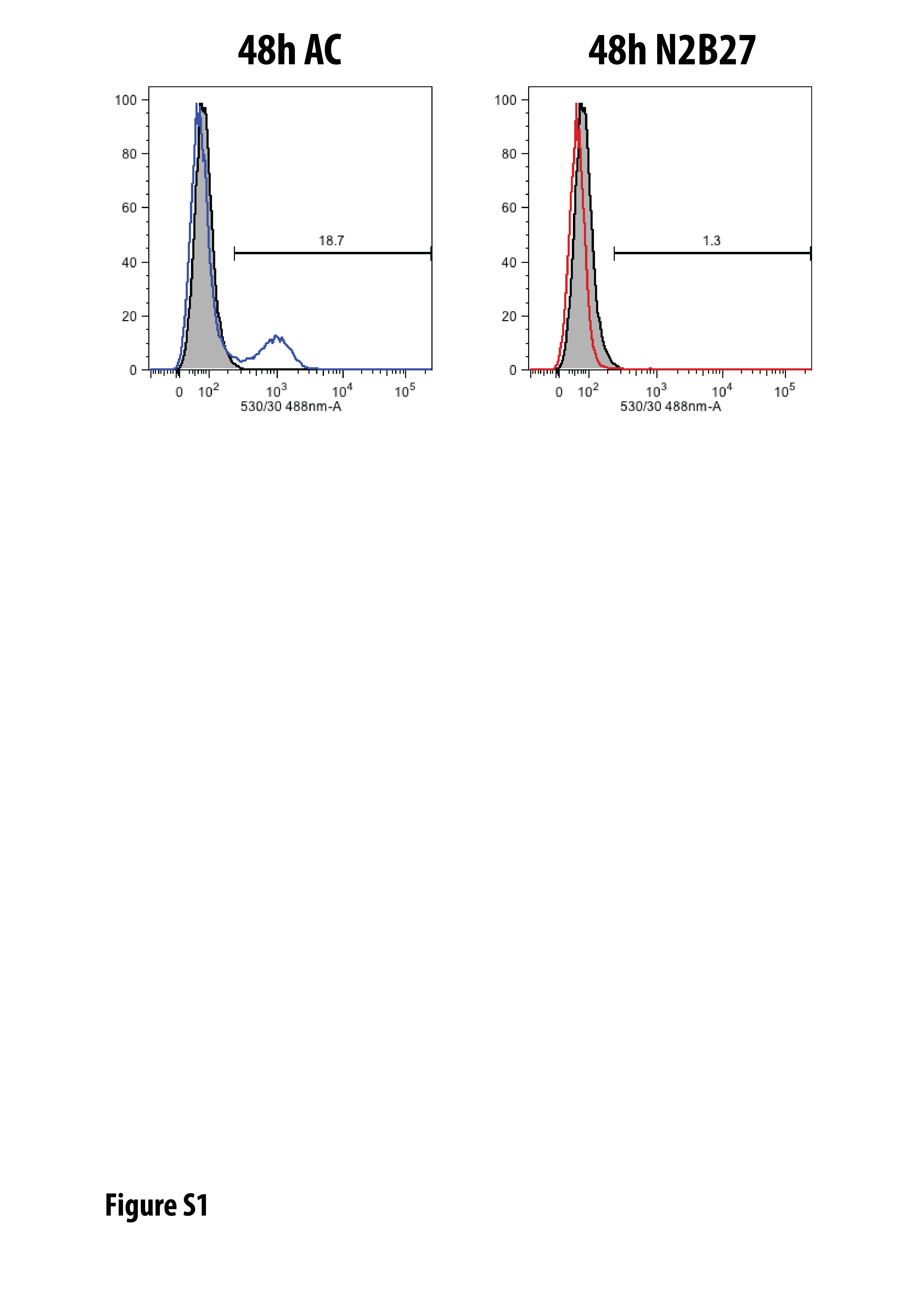

Supplement: Additional file 1: Figure S1. — Cells cultured in N2B27 are unable to express the Bra-GFP reporter. Bra::GFP mESCs were plated and differentiated for 48 h in either N2B27 supplemented with Act/Chi (positive control; blue line left panel) or in N2B27 (red line, right panel) without any other factors. The percentage of GFP-positive cells for Act/Chi or N2B27 conditions are shown. An E14-Tg2A WT control is displayed in each panel (tinted grey profile). AC, activin A + chiron; Bra, brachyury; GFP, green fluorescent protein; WT, wild type. [file 12915_2014_63_MOESM1_ESM.jpeg]

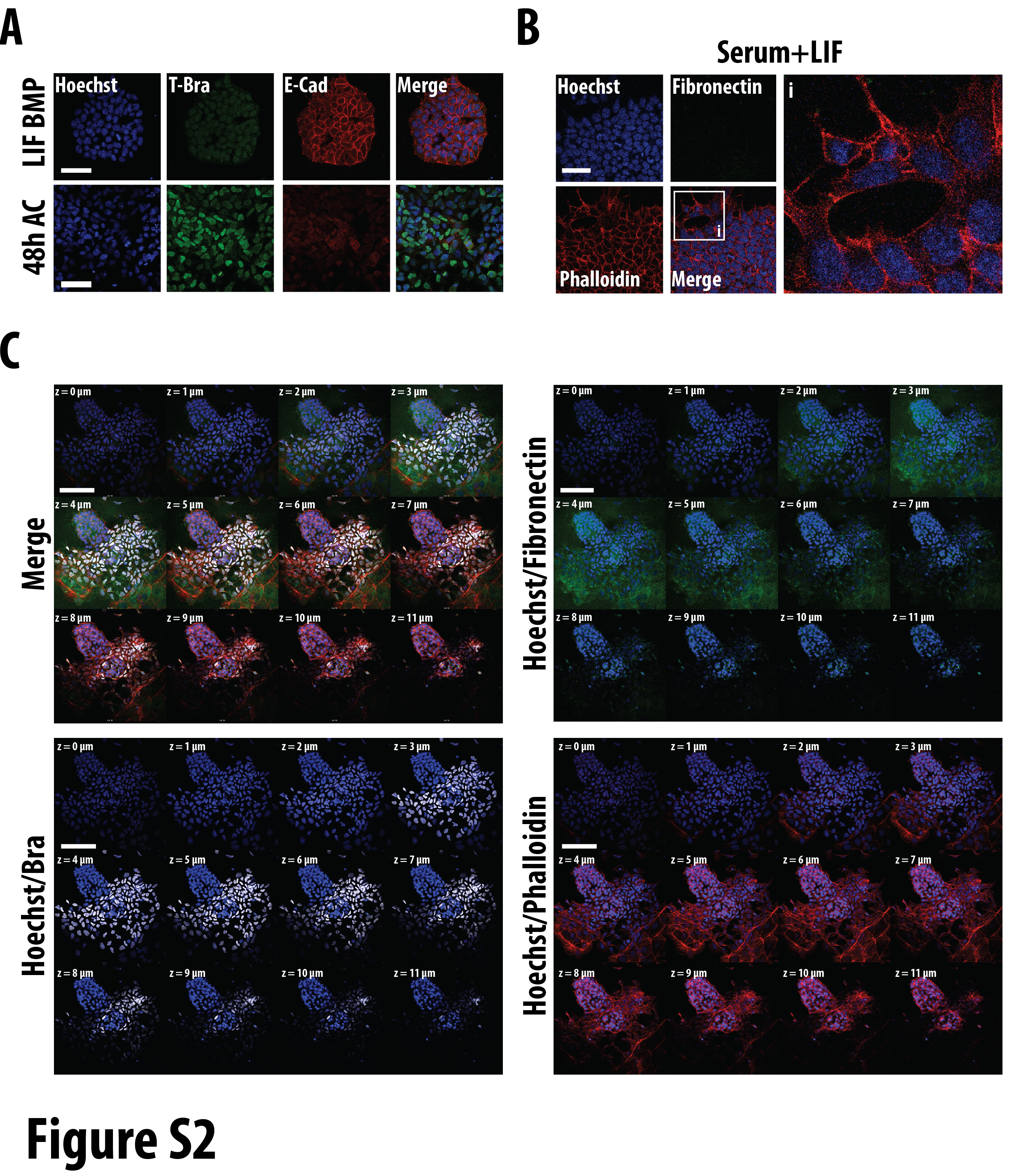

Supplement: Additional file 2: Figure S2. — Status of brachyury, E-cadherin, fibronectin and F-actin in pluripotent and differentiating E14-Tg2A mESCs. (A) E14-Tg2A mESCs in LB or differentiated in Act and Chi for 48 h, stained for Bra (green) and E-cadherin (E-Cad; red) and imaged by confocal microscopy. Act and Chi treatment alters Bra expression and causes a loss of E-cadherin from the membrane. (B) E14-Tg2A mESCs cultured in serum and LIF, stained for fibronectin (green) and with phalloidin to mark F-actin (red). The pluripotent state is characterized by low fibronectin and F-actin localized to cell–cell boundaries, lamellipodia and protruding filopodia; see magnified region (i). (C) A montage illustrating the z-section (in 1 μm increments) of the colony imaged in Figure 2C and Figure S2D showing all fluorescent channels merged (top left), Hoechst stain and fibronectin (top right), Hoechst stain and Bra (bottom left) and Hoechst stain and phalloidin (bottom right). Hoechst stain, fibronectin, brachyury and phalloidin are coloured blue, green, white and red respectively. See main text for details. Hoechst stain was used to mark the nuclei. Scale bars indicate 50 μm in (A) and (B), and 100 μm in (C). Act, activin A; BMP, bone morphogenetic factor; Bra, brachyury; Chi, chiron CHIR99021; E-Cad, E-cadherin; LB, leukaemia inhibitory factor and bone morphogenetic factor; LIF, leukaemia inhibitory factor. [file 12915_2014_63_MOESM2_ESM.jpeg]

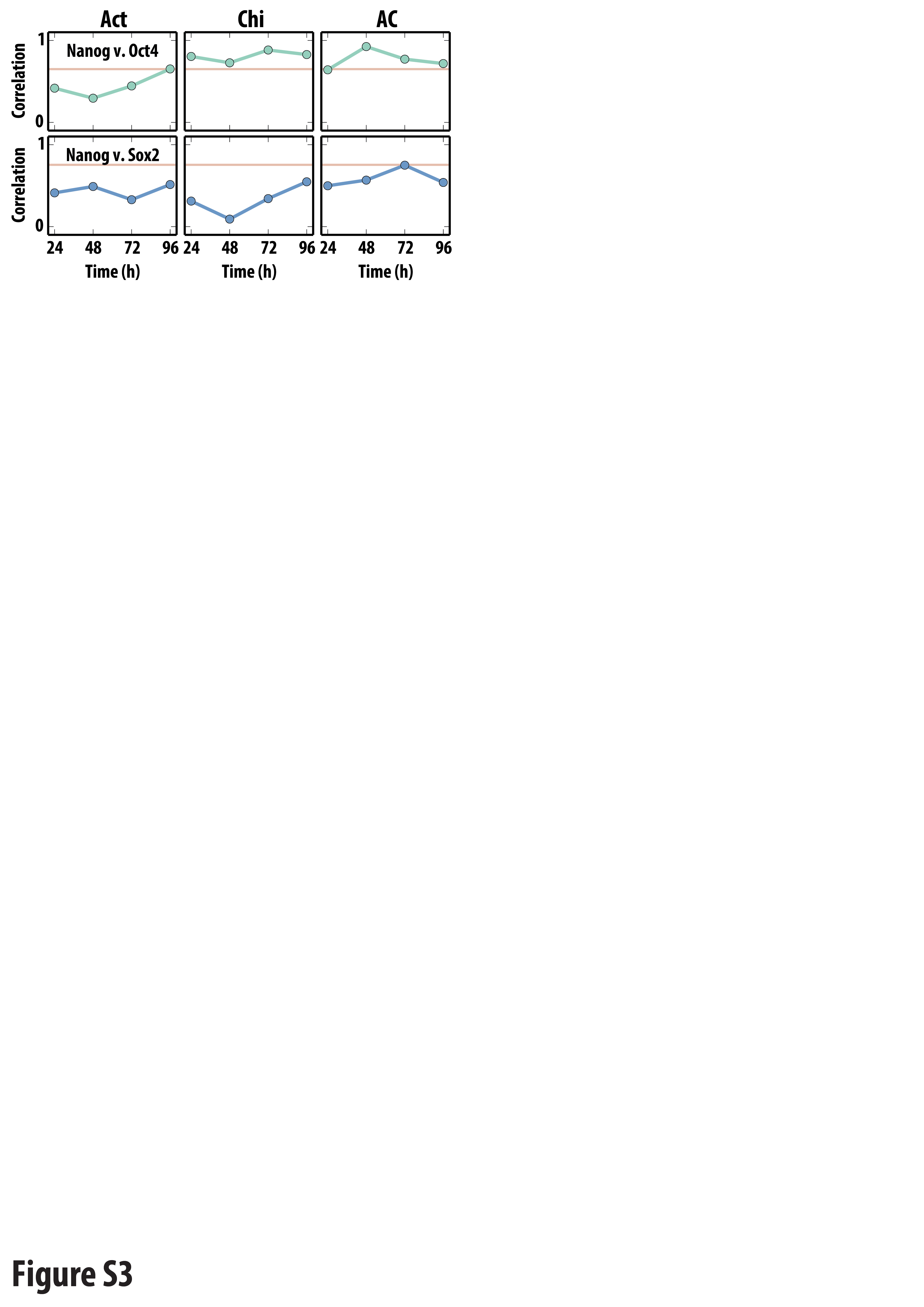

Supplement: Additional file 3: Figure S3. — Pearson correlation coefficients. Values for Nanog and Oct4 (top), Nanog and Sox2 (bottom) for the different time points for Act, Chi and Act/Chi. The horizontal line represents the correlation for LB. Differentiation produces strong correlations between Nanog and Oct4 conditions whereas the correlation between Nanog and Sox2 increases with time. AC, activin A + chiron; Act, activin A; Chi, chiron CHIR99021; LB, leukaemia inhibitory factor and bone morphogenetic factor. [file 12915_2014_63_MOESM3_ESM.jpeg]

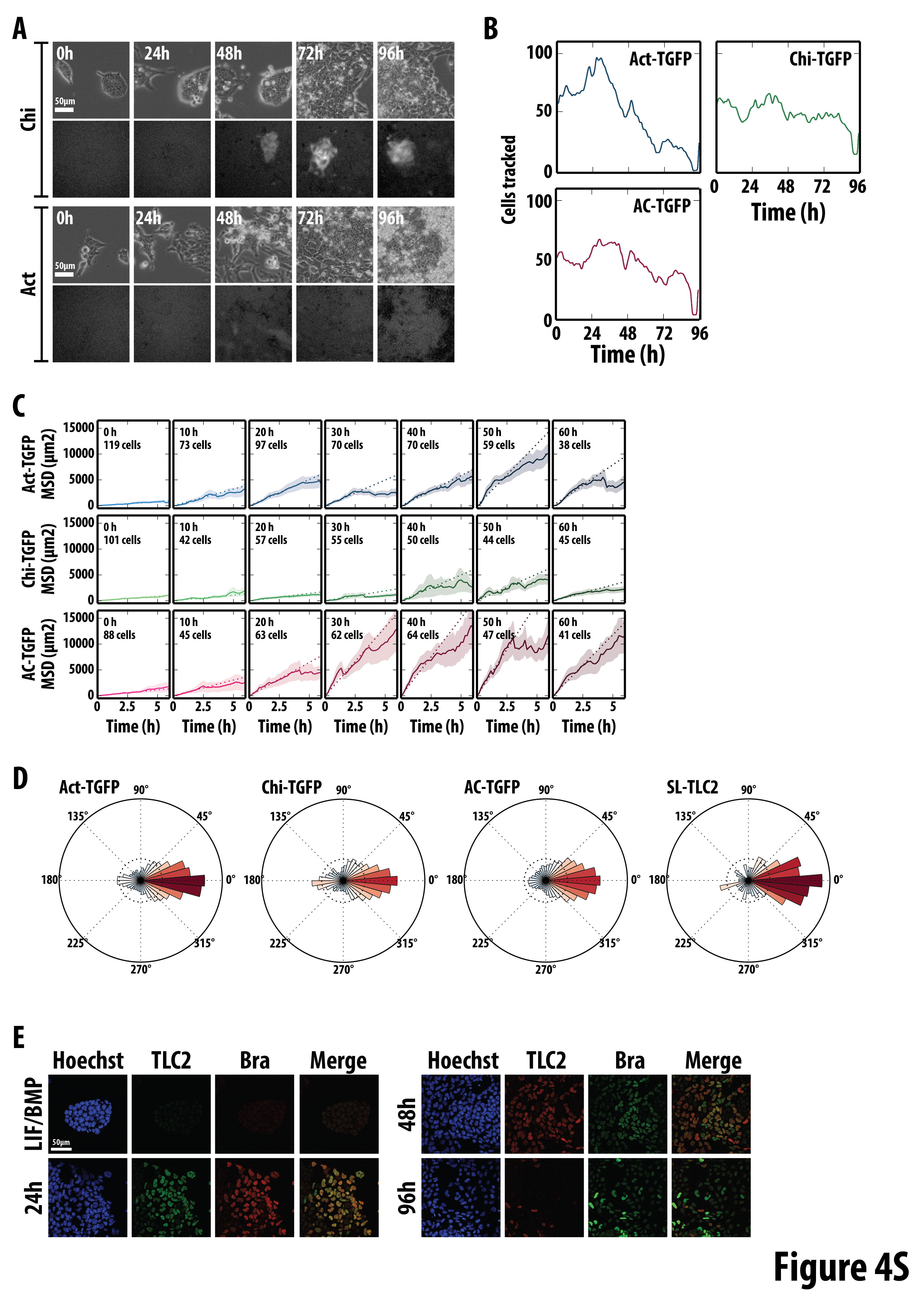

Supplement: Additional file 4: Figure S4. — Wnt/β-catenin transcriptional reporter (TLC2) following mesodermal differentiation. (A) Still images from live imaging of Bra::GFP mESCs in Act/Chi conditions (Additional file 5: Movie M1) showing phase contrast (top rows) and fluorescence (bottom rows). Act treatment results in lower levels of fluorescence compared with Chi or Act/Chi (Figure 4). (B) The number of cells tracked per condition over time. (C) The mean square displacement (MSD) of individual cells. Cell traces were separated into 10-h time intervals based on the time of tracking initiation. The number of cells within each time interval is displayed. The start of each cell trace within each time interval is aligned. See text for details. (D) Distribution of turning angles for all cells treated with Act, Chi and Act/Chi. A serum-LIF control from the TLC2 reporter cell line is included for comparison. There appears to be no bias in the direction cells move with respect to the conditions in which they are placed. (E) TLC2 reporter cells fixed at the indicated times and stained for Bra. Scale bar in all images denotes 50 μm. AC, activin A + chiron; Act, activin A; BMP, bone morphogenetic factor; Bra, brachyury; Chi, chiron CHIR99021; SL, serum leukaemia inhibitory factor; MSD, mean-squared displacement. [file 12915_2014_63_MOESM4_ESM.jpeg]

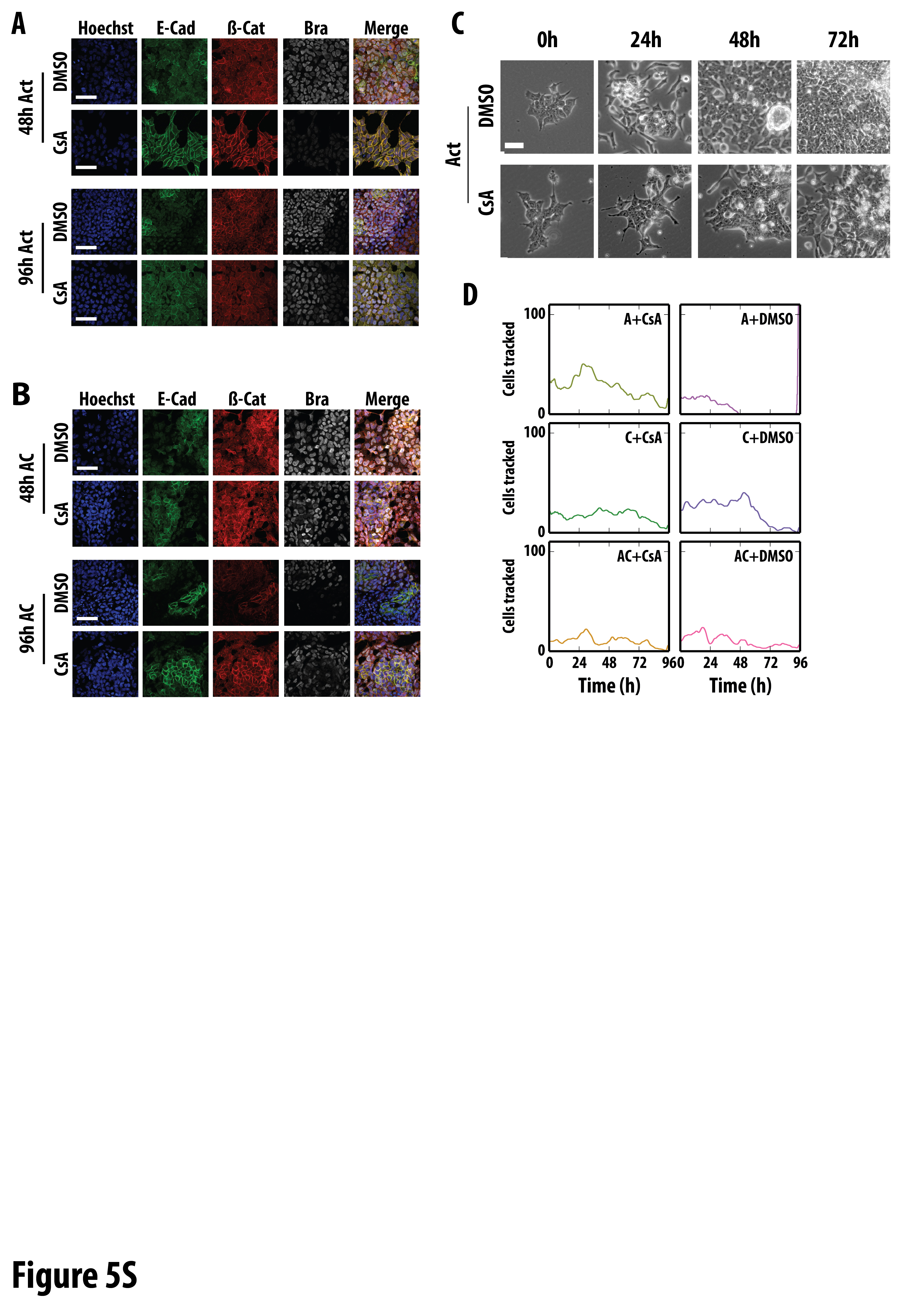

Supplement: Additional file 7: Figure S5. — An EMT event is required for brachyury expression. (A,B) E14-Tg2A mESCs treated with Act (A) or Act/Chi (B) in the presence of CsA (3 μM) or DMSO for 48 and 96 h and stained for Hoechst, E-cadherin, β-catenin and Bra. In the presence of CsA, E-cadherin is not effectively cleared from the membrane, β-catenin does not enter the nucleus and there is no effective expression of Bra. (C) Stills from live imaging of E14-Tg2A mESCs in Act with DMSO or CsA. As with Chi (Figure 5C) cells in CsA stretch out filopodia but do not undergo an EMT. (D) The number of cells tracked in each condition over time. Scale bar in all images denote 50 μm. AC, activin A + chiron; Act, activin A; β-cat, β-catenin; Bra, brachyury; Chi, chiron CHIR99021; CsA, cyclosporine A; DMSO, dimethyl sulfoxide; E-Cad, E-cadherin; EMT, epithelial-to-mesenchymal transition; mESC, mouse embryonic stem cell. [file 12915_2014_63_MOESM7_ESM.jpeg]

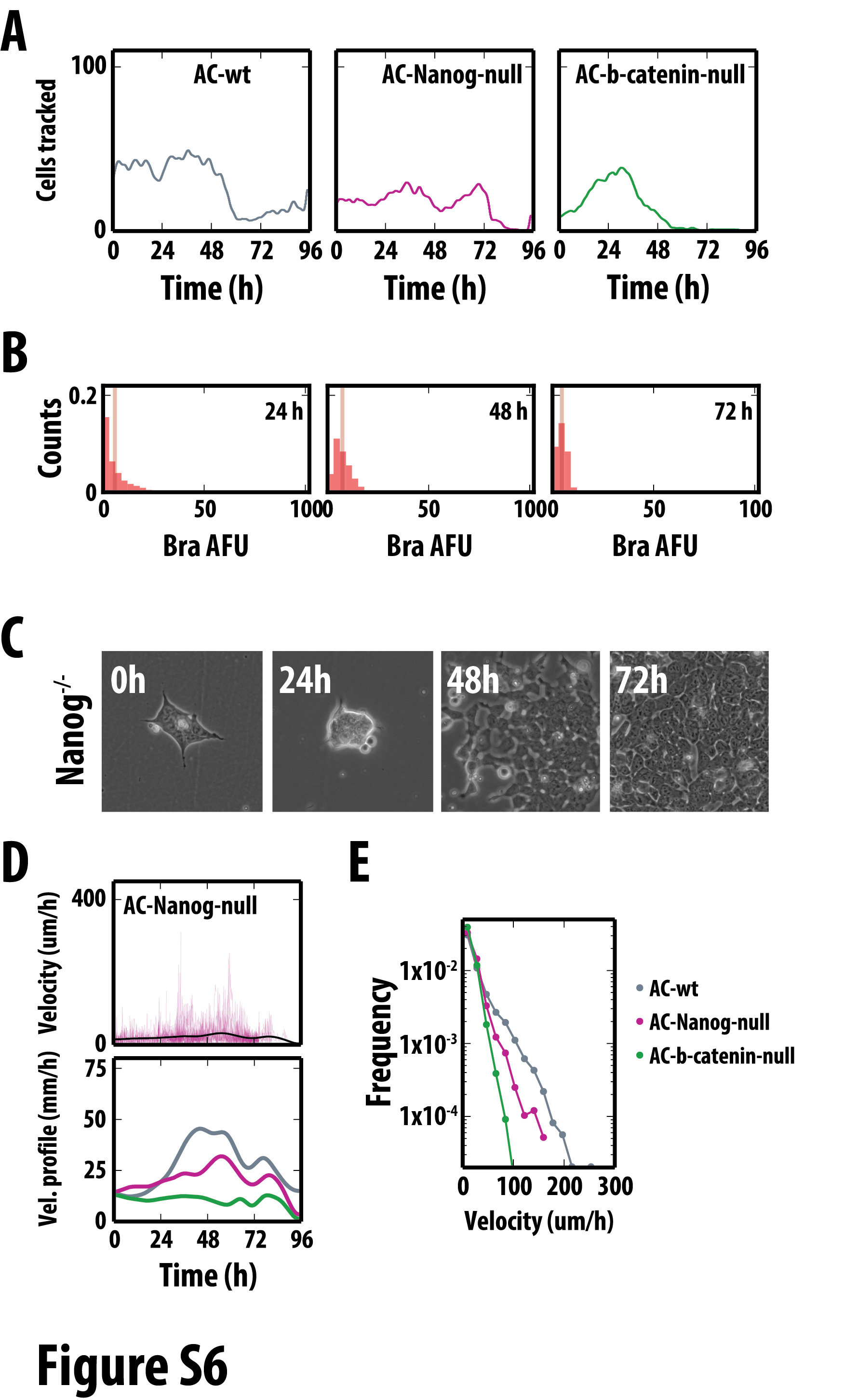

Supplement: Additional file 8: Figure S6. — Immunofluorescence analysis and live-cell imaging of Nanog-/- mESCs following differentiation. (A) The number of cells tracked in each condition over time for WT, Nanog−/− and β-catenin−/− mESCs. (B) Time evolution of the distributions of the expression of Bra in Nanog−/− mESCs. The cells, treated with Act/Chi for 24, 48 and 72 h, were stained for Bra and nuclei were segmented based on Hoechst staining. The average pixel intensity for each fluorescent channel was quantified and the intensity of Bra displayed as histograms for each time point. The bisecting orange lines in each histogram correspond to the mean fluorescence levels. (C) Live imaging of Nanog mutants in Act/Chi. (D) Individual cell velocities (μm/h), and the average velocity profile for each cell line (indicated by colours) in Act/Chi. (E) The distribution of velocities over time. For comparison, the velocities and distribution of velocities for the WT and β-catenin mutant cell lines (Figure 6) are included in (D) and (E). AC, activin A + chiron; Act, activin A; AFU, arbitrary fluorescence units; Bra, brachyury; Chi, chiron CHIR99021; mESC, mouse embryonic stem cell; Vel., velocity; WT, wild type. [file 12915_2014_63_MOESM8_ESM.jpeg]

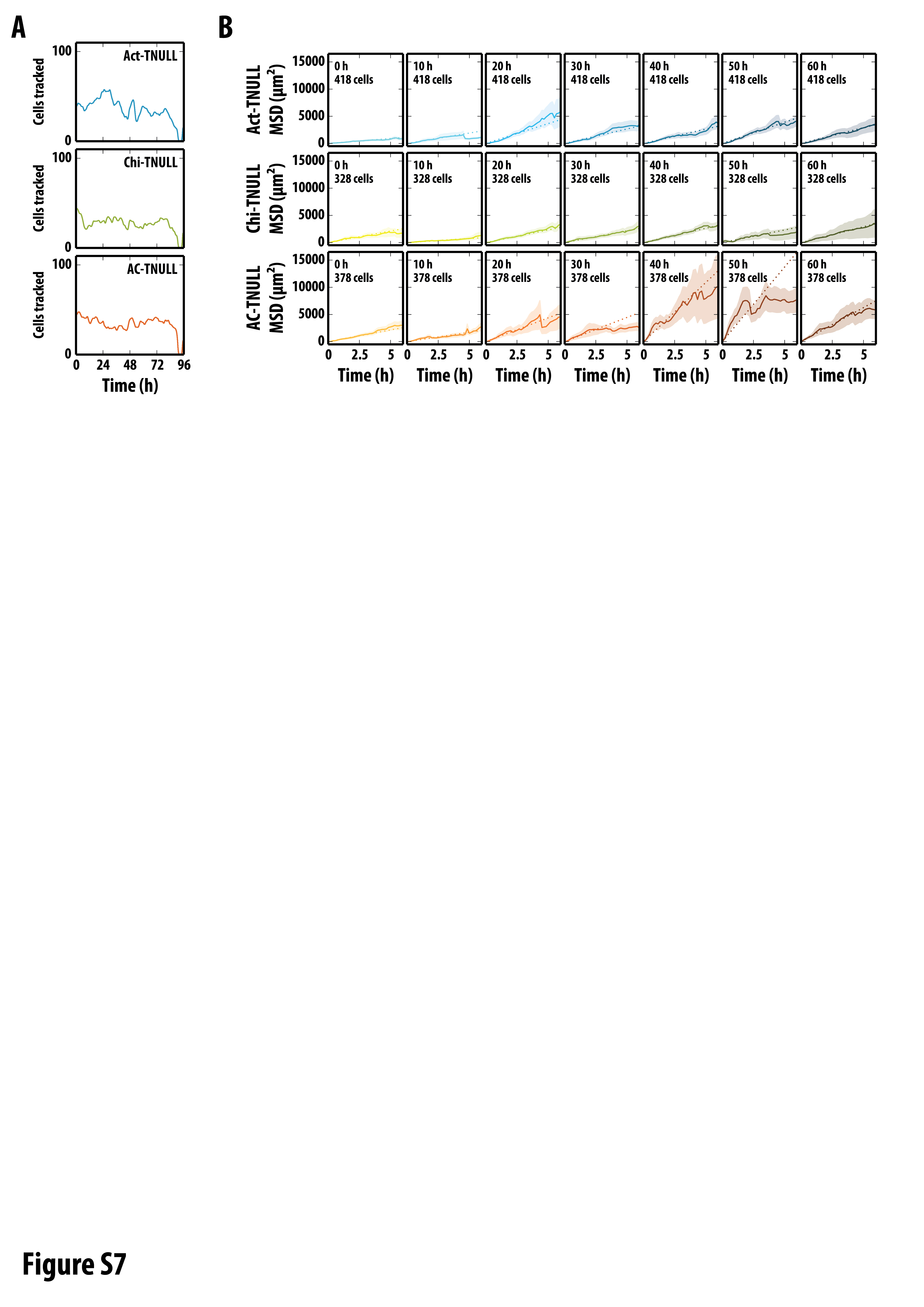

Supplement: Additional file 9: Figure S7. — Analysis of individual Bra-/- cells from live-imaging experiments following Act, Chi and Act/Chi stimulation. (A) The number Bra−/− mESCs tracked from live-cell imaging (Figure 7) in each condition over time. (B) The mean square displacement (MSD) of individual cells. Cell traces were separated into 10-h time intervals based on the time of tracking initiation. The number of cells within each time interval is displayed. The start of each cell trace within each time interval is aligned. See text for details. AC, activin A + chiron; Act, activin A; Bra, brachyury; Chi, chiron CHIR99021; mESC, mouse embryonic stem cell; MSD, mean-squared displacement. [file 12915_2014_63_MOESM9_ESM.jpeg]
