# Supplementary material for: Brachyury cooperates with Wnt/β-catenin signalling to elicit primitive-streak-like behaviour in differentiating mouse embryonic stem cells
Source: BMC Biol. 2014 Aug 13;12:63. doi: 10.1186/s12915-014-0063-7 (PMC4171571; doi:10.1186/s12915-014-0063-7)
Supplement: Additional file 10: Table S1. — Summary of the tracking data. For each cell line used in the live-cell imaging experiments within this investigation (leftmost column: cell type), the number of movies analysed, the temporal resolution (frames/h), spatial resolution (pixels/μm), the number of cells tracked, positions tracked and the average number of positions per cell for each medium condition (second column from the left; medium conditions) to which the cells were exposed was tabulated. The total number of movies analysed and the total number of cells tracked throughout the investigation are recorded at the bottom of the table. This table can be used in conjunction with the number of cells tracked over time, displayed in Additional file 4: Figure S4B, Additional file 7: Figure S5D, Additional file 8: Figure S6A and Additional file 9: Figure S7A. [file 12915_2014_63_MOESM10_ESM.doc]

**Supplementary Table and Legend**

**Table S1: Summary of the tracking data collected.** For each cell line used in the live-cell imaging experiments within this investigation (left most column; ‘cell type’), the information relating to the number of movies analysed, the temporal resolution (frames/h), spatial resolution (pixels/µm), the number of cells tracked, positions tracked and the average number of positions per cell for each medium condition (second column from the left; Medium conditions) to which the cells were exposed was tabulated. The total number of movies analysed and the total number of cells tracked throughout the investigation is recorded at the bottom of the table. This table can be used in conjunction with the number of cells tracked over time, displayed in Figs. S4B, S5D, S6A and S7A.

| Cell type | Medium conditions | # movies | Temporal resolution (frames/h) | Spatial resolution (px/µm) | # cells tracked | # positions tracked | Avg. positions per cell |
| --- | --- | --- | --- | --- | --- | --- | --- |
| Bra::GFP | Act | 20 | 6 | 1.034 (Movies 1-9), 1.55 (Movies 10-20) | 583 | 28,102 | 48.2 |
|  | Chi | 20 | 6 |  | 497 | 28,190 | 56.7 |
|  | AC | 20 | 6 |  | 501 | 26,199 | 52.3 |
| Bra::GFP | Act+DMSO | 3 | 4 | 1.55 | 97 | 3,195 | 32.9 |
|  | Chi+DMSO | 4 | 4 | 1.55 | 238 | 3,799 | 16.0 |
|  | AC+DMSO | 2 | 4 | 1.55 | 141 | 3,946 | 28.0 |
|  | Act+CsA | 4 | 4 | 1.55 | 263 | 2,345 | 8.9 |
|  | Chi+CsA | 3 | 4 | 1.55 | 141 | 6,564 | 46.6 |
|  | AC+CsA | 2 | 4 | 1.55 | 115 | 3,912 | 34.0 |
| Bra^-/-^ | Act | 9 | 6 | 1.55 | 418 | 20,149 | 48.2 |
|  | Chi | 9 | 6 | 1.55 | 328 | 15,277 | 46.6 |
|  | AC | 9 | 6 | 1.55 | 378 | 19,343 | 51.2 |
| TLC2 | AC | 2 | 4 | 1.55 | 127 | 3,711 | 29.2 |
| Nanog^-/-^ | AC | 4 | 4 | 1.55 | 192 | 4,782 | 24.9 |
| E14-Tg2A^+/+^ | AC | 3 | 4 | 1.55 | 372 | 1,757 | 4.7 |
| β-catenin-/- | AC | 2 | 4 | 1.55 | 145 | 1,324 | 9.1 |
| (Totals) |  | 116 |  |  | 4,536 | 172,595 | 38.1 |
